# Matters of life and breath: A role for hypoxia in determining cell state

**DOI:** 10.18632/aging.100480

**Published:** 2012-08-20

**Authors:** Marco Demaria, Judith Campisi

**Affiliations:** Buck Institute for Research on Aging, Novato CA 94945, USA

Mammalian cells require oxygen for essentially all aspects of metabolism during both embryonic development and adult life [1]. Yet, *in vivo*, mammalian cells experience a wide range of oxygen concentrations, depending on the tissue type, proximity of cells to major arterioles and capillaries, and pathological conditions such as blood vessel occlusions, other sources of ischemia and neoplasia. Cells have therefore evolved programmed responses to very low oxygen concentrations, or hypoxia, which helps them withstand hypoxic environments. Hypoxic responses have traditionally been view as survival mechanisms that are essential for the proper function of cells that reside in naturally low oxygen tissue environments -- and for the survival of cells challenged by pathological ischemia. Now, new findings by the Blagosklonny laboratory [2] uncover a novel, non-canonical role for hypoxia in determining whether cells enter a state of quiescence or senescence. These findings have particular importance for certain stem cells, such as those in the relatively hypoxic niches of the bone marrow or tumor masses, and hence for a variety of diseases [3], especially those associated with aging.

The Blagosklonny group used a cancer cell line that ceases division upon inducible, ectopic expression of the cell cycle inhibitor p21. They previously showed that this induced expression rapidly arrests cell proliferation. The arrest is reversible for 2-3 days – that is, by all criteria, it would be classified as quiescence, the reversibly arrested state in which many cells reside *in vivo*. Thereafter, however, the arrest becomes irreversible and closely resembles the state termed cellular senescence. Senescence is a double-edged sword [4]. It protects organisms from cancer by arresting the growth of cells at risk for neoplastic transformation, and may be beneficial under some conditions of tissue injury. But the senescence response also entails a senescence-associated secretory phenotype (SASP): the secretion of numerous pro-inflammatory cytokines. The SASP can fuel chronic inflammation -- a major cause or contributor to many age-related pathologies, including cancer [4]. Thus, the ectopic p21 expression causes cells to switch between two distinct states: initially, it induces quiescence, which converts to senescence after a few days. The Blagonsklonny group terms this process geroconversion.

The new paper [2] shows that under hypoxia -- 0.2% oxygen, which is significantly below the oxygen levels found in most, though not all, normal tissues [1] – ectopic p21 expression failed to cause geroconversion. That is, hypoxia prevented the conversion from quiescence to senescence. This finding was not limited to the p21-inducible cancer cells; it was also seen in immortal but non-tumorigenic human fibroblasts and normal human retinal pigment epithelial (RPE) cells, both induced to senesce by the DNA damaging agent etoposide. It is significant that hypoxia protected RPE cells from geroconversion: the senescence of these cells *in vivo* is thought to cause or contribute to age-related macular degeneration [5].

Of particular interest, hypoxia resembled the effects of rapapmycin, a macrolide antibiotic that is currently in clinical use, mainly to prevent organ transplant rejection. Rapamycin is a potent inhibitor of selected activities of the mTOR protein kinase, which defines an important pathway that drives aging in diverse eukaryotic organisms [6]. The Blagosklonny group previously showed that rapamycin also inhibits geroconversion [7]. Thus, their recent findings strongly suggest that rapamycin and hypoxia converge on the same pathway and mechanism – inhibition of mTOR activity. Consistent with this hypothesis, hypoxia, much like rapamycin, suppressed phosphorylation of the S6 ribosomal protein, a major mTOR substrate.

The effects of hypoxia in inhibiting geroconversion were independent of p53, which can also inhibit the mTOR pathway. Remarkably, the effects were also independent of HIF-1, the transcription factor that is responsible for inducing the majority of hypoxia-induced changes in gene expression. This finding is in contrast to the effects of HIF-1 under normoxic or hyperoxic conditions; under those conditions and in mouse fibroblasts, HIF-1 delays replicative senescence, largely through the induction of macrophage inhibitory factor (MIF) [8]. Thus, hypoxia appears to inhibit the transition from quiescence to senescence by a non-canonical mechanism that is independent of both p53 and HIF-1.

What is the physiological significance of the findings that hypoxia, like rapamycin, inhibits the quiescence to senescence conversion? One interesting possibility is that these findings define a mechanism by which stem cells that reside in a relatively hypoxic environment are able to survive and, importantly, persist in a quiescent state in order to be available for tissue repair and regeneration. This protective effect of hypoxia may be particularly important for incidences of severe genotoxic stress, such as that experienced by patients undergoing radio- or chemo-therapy or survivors of environmental disasters. In these cases, it would be advantageous to protect stem cells from senescence – a common response to genotoxic stress [4] –to ensure their availability for tissue repair and regeneration during recovery from the stress. On the negative side, these new findings suggest hypoxia can also protect cancer stem cells from undergoing senescence in response to genotoxic therapies, and thus increase the probability of cancer recurrence. In either case, these intriguing findings open the way for developing strategies to either stimulate or prevent senescence by manipulating the oxygenation state of the tissue environment.
